# ﻿Two novel species and a new host record of *Alternaria* (Pleosporales, Pleosporaceae) from sunflower (Compositae) in Myanmar

**DOI:** 10.3897/mycokeys.105.123790

**Published:** 2024-06-07

**Authors:** Zin Mar Nwe, Khin Nayyi Htut, Sein Lai Lai Aung, Ya-Nan Gou, Cheng-Xin Huang, Jian-Xin Deng

**Affiliations:** 1 Department of Plant Protection, College of Agriculture, Yangtze University, Jingzhou 434025, China; 2 MARA Key Laboratory of Sustainable Crop Production in the Middle Reaches of the Yangtze River (Co-Construction by Ministry and Province), Yangtze University, Jingzhou 434025, China; 3 Department of Plant Pathology, Yezin Agricultural University, Nay Pyi Taw, Myanmar

**Keywords:** *Alternaria*, morphology, new host record, novel species, phylogeny

## Abstract

Sunflower (*Helianthusannuus* L.) is a widely cultivated, fast-growing crop known for its seeds and oil, with substantial ecological and economic importance globally. However, it faces challenges from leaf diseases caused by *Alternaria* species, which threaten its yield. Three small-spored *Alternaria* species were isolated from leaf spot and blight symptoms on sunflower in Myanmar. All the species were determined based on morphological characterization and a multi-locus phylogenetic assessment of seven genes, including the internal transcribed spacer of rDNA region (ITS), glyceraldehyde-3-phosphate dehydrogenase (*GAPDH*), RNA polymerase second largest subunit (RPB2), translation elongation factor 1-α (*TEF1*), *Alternaria* major allergen gene (*Alt a 1*), endopolygalacturonase gene (*EndoPG*), and an anonymous gene region (OPA10-2). The results introduced two new *Alternaria* species, *A.myanmarensis***sp. nov.** and *A.yamethinensis***sp. nov.**, and a known species of *A.burnsii*, firstly reported from sunflower.

## ﻿Introduction

The genus *Alternaria* Nees, 1816, which belongs to the family Pleosporaceae, order Pleosporales, and phylum Ascomycota, is a widely distributed dematiaceous fungus frequently found in plants, soil, food, and indoor air environments (Thomma 2003). It includes more than 790 species epithets, and approximately 382 species have been accepted (Hongsanan et al. 2020; Wijayawardene et al. 2020; Gannibal et al. 2022; Li et al. 2023; Liao et al. 2023). The identification and classification of *Alternaria* commonly rely on cultural features, conidial characteristics (shape, size, septation, beak formation), sporulation patterns, and hosts (Zhang 2003; Simmons 2007; Yu 2015). Normally, *Alternaria* is categorized into two obviously distinct groups: large-spored and small-spored *Alternaria* (Simmons 2007). The conidial bodies of large-spored species typically measure 60–100 μm in length and the small-spored species are less than 60 μm. The morphological criteria can be influenced by growth conditions, including substrate, light, and humidity, potentially undermining their reliability in characterizing the genus (Woudenberg et al. 2013).

Nowadays, diverse molecular techniques have been utilized to clarify the variability among and within *Alternaria* species (Lawrence et al. 2016). The classification has been significantly informed through phylogenetic analysis by utilizing more than ten distinct genetic loci. These loci include the regions of rDNA (nuclear small subunit (*SSU*), large subunit (*LSU*), and internal transcribed spacer (ITS)), glyceraldehyde-3-phosphate dehydrogenase (*GAPDH*), RNA polymerase second largest subunit (*RPB2*), translation elongation factor 1-α (*TEF1*), *Alternaria* major allergen (*Alt a 1*), endopolygalacturonase (*EndoPG*), an anonymous genomic region (OPA10-2), calmodulin (*CAL*), and eukaryotic orthologous groups (KOG) (Liu et al. 2024). The genus is found to encompass 29 sections through comprehensive multi-locus phylogenetic analyses (Ghafri et al. 2019; Gannibal et al. 2022). Among them, the section Alternaria, which includes members with catenate and small-spored conidia, is recognized as having only 11 phylogenetic species and one species complex (Woudenberg et al. 2015).

Leaf spot and blight disease on sunflower (*Helianthusannuus* L.) caused by *Alternaria* significantly decreases head diameters and seed production (Kgatle et al. 2020). Sunflower, belonging to the Compositae family and native to North America, is an oilseed crop cultivated worldwide, with its oil ranking as the second most important source of edible vegetable oils (Zhang et al. 2021). The plant is also commercialized for livestock feed (Yegorov et al. 2019). It was introduced to Myanmar in 1968 (Favre and Myint 2009) and covered 0.224 million hectares with a yield of 9245 kg/ha in 2022 (http://faostat.fao.org/site/567/default.aspx#ancor). In the Central Dry Zone of Myanmar (Mandalay, Sagaing, and Magway Regions), it contributes to more than 77% of the overall oilseed crop production (DOA 2020). During the monsoon season in 2023, three small-spored *Alternaria* species were isolated from leaf symptoms of sunflower collected in a plantation in Mandalay, Myanmar. In this study, those species were meticulously identified and illustrated through morphological and phylogenetic approaches.

## ﻿Materials and methods

### ﻿Sample collection and fungal isolation

In August 2023, sunflower leaves displaying spot and blight symptoms were randomly collected from plantations in Myanmar, Mandalay Region, Yamethin Township, Segyi Village (30°21'28.188"N, 112°08'32.136"E). From each field, samples were randomly collected at five different points, placed in separate clean zip bags and transported to the laboratory. For fungal isolation, leaf fragments from the edges of the lesions were excised, treated with a 1% sodium hypochlorite solution for three minutes, rinsed three times with distilled water, plated on moist filter papers in Petri dishes and then incubated at 25 °C in the dark for sporulation. A single spore was picked using a sterile glass needle under a stereomicroscope and inoculated onto potato dextrose agar (PDA: Difco, Montreal, Canada). Once sufficiently grown, pure cultures were isolated by a single spore and preserved in test tube slants at 4 °C in the Fungi Herbarium at Yangtze University (YZU) in Jingzhou, Hubei, China. MycoBank numbers were obtained by following the protocols outlined on (https://www.mycobank.org/).

### ﻿Morphological characterization

To study the characteristics of the culture, mycelial plugs (6 mm diameter) were extracted from the periphery of 5-day-old colonies growing on PDA, transferred to fresh 90 mm PDA plates, and incubated in darkness at 25 °C for 7 days. For the examination of conidial morphology, mycelia were cultured on V8 juice agar (V8A) and potato carrot agar (PCA) under white fluorescent light at 22 °C with an 8-hour light/16-hour dark period (Simmons 2007). After a 7-day incubation period, the sporulation patterns and conidial characteristics were determined under an ECLIPSE Ni-U microscopic system (Nikon, Japan). The conidia were observed using a lactophenol-picric acid solution. Fifty randomly selected conidia were recorded.

### ﻿DNA extraction, PCR amplification, and Sequencing

Genomic DNA extraction involved scraping fresh mycelia from colonies cultivated on PDA for 5 days at 25 °C, following the method outlined by Watanabe et al. (2010). Polymerase chain reaction (PCR) amplification and sequencing targeted specific genes of the internal transcribed spacer region of rDNA (ITS), glyceraldehyde-3-phosphate dehydrogenase (*GAPDH*), RNA polymerase second largest subunit (*RPB2*), translation elongation factor 1-α (*TEF1*), *Alternaria* major allergen (*Alt a 1*), endopolygalacturonase gene (*EndoPG*), and an anonymous genomic region (OPA10-2). In the PCR processes, a 25 μL reaction mixture was prepared, consisting of 21 μL of 1.1× Taq PCR Star Mix (TSINGKE), 2 μL of template DNA, and 1 μL of each primer. The amplification reaction was performed using a Bio-Rad T100 thermocycler according to the conditions listed in (Table 1). The generated products underwent electrophoresis in a 1% agarose gel and were visualized by UV transillumination. Subsequently, the amplified products were purified and sequenced in both directions, facilitated by TSINGKE Company (Beijing, China). Initially, sequences from both ends were examined and manually edited using BioEdit v. 7.0.9 (Hall 1999). Following this, the sequences were aligned and further edited with the PHYDIT v3.2 software (Chun 1995) before being submitted to GenBank (https://www.ncbi.nlm.nih.gov/) (Table 2).

**Table 1.** Primers and PCR protocols.

Gene regions	Primers	PCR conditions	References
ITS	ITS5/ITS4	94 °C for 3 min, 34 cycles of 94 °C for 30 s, 55 °C for 30 s and 72 °C for 2 min, 72 °C for 10 min	White et al. 1990
*GAPDH*	gpd1/gpd2	95 °C for 2 min, 32 cycles of 95 °C for 30 s, 56 °C for 30 s and 72 °C for 42 s, 72 °C for 5 min	Berbee et al. 1999
*RPB*2	RPB2-5F/ RPB2-7cR	94 °C for 5 min, 34 cycles of 94 °C for 45 s, 57 °C for 45 s and 72 °C for 1 min, 72 °C for 10 min	Sung et al. 2007
*TEF1*	EF1-728F/ EF1-986R	94 °C for 3 min, 35 cycles of 94 °C for 30 s, 55 °C for 45 s and 72 °C for 1 min, 72 °C for 10 min	Carbone and Kohn et al. 1999
*Alt a 1*	Alt-for/ Alt-rev	94 °C for 2 min, 33 cycles of 94 °C for 30 s, 60 °C for 30 s and 72 °C for 30 s, 72 °C for 10 min	Hong et al. 2005
*EndoPG*	PG3/ PG2b	94 °C for 3 min, 33 cycles of 94 °C for 30 s, 50 °C for 30 s and 72 °C for 59 s, 72 °C for 5 min	Andrew et al. 2009
OPA10-2	OPA10-2L/ OPA10-2R	94 °C for 2 min, 33 cycles of 94 °C for 30 s, 56 °C for 30 s and 72 °C for 30 s, 72 °C for 10 min	Andrew et al. 2009

**Table 2.** The GenBank accession numbers of *Alternaria* strains used in the present study.

Species	Strain	Host/Substrate	Country	GenBank accession numbers						
				ITS	*GAPDH*	*TEF1*	*RPB2*	*Alt a 1*	*Endo-PG*	OPA10-2
*A.alternantherae*	CBS 124392	*Solanummelongena*	China	KC584179	KC584096	KC584633	KC584374	KP123846	–	–
*A.alternata*	CBS 916.96^T^	*Arachishypogaea*	India	AF347031	AY278808	KC584634	KC584375	AY563301	JQ811978	KP124632
	CBS 102604	*Minneolatangelo*	Israel	KP124334	AY562410	KP125110	KP124802	AY563305	KP124035	KP124643
	CBS 102596	*Citrusjambhiri*	USA	KP124328	KP124183	KP125104	KP124796	KP123877	KP124030	KP124637
	CBS 918.96	*Dianthuschinensis*	UK	AF347032	AY278809	KC584693	KC584435	AY563302	KP124026	KP124633
	CBS 106.34	*Linumusitatissimum*	Unknown	Y17071	JQ646308	KP125078	KP124771	KP123853	KP124000	KP124608
	CBS 121547	*Pyrusbretschneideri*	China	KP124372	KP124224	KP125150	KP124842	KP123920	KP124076	KP124685
	CBS 101.13	Peat soil	Switzerland	KP124392	KP124244	KP125170	KP124862	KP123940	KP124096	KP124705
	CBS 126.60	Wood	UK	KP124397	KP124249	KP125175	KP124867	JQ646390	KP124101	KP124710
	CBS 109730	*Solanumlycopersicum*	USA	KP124399	KP124251	KP125177	KP124869	KP123946	KP124103	KP124713
	CBS 119545^T^	*Senecioskirrhodon*	New Zealand	KP124409	KP124260	KP125187	KP124879	KP123956	KP124113	KP124723
*A.baoshanensis*	MFLUCC 21-0124^T^	*Curcubitamoschata*	China	MZ622003	OK236706	OK236613	OK236659	OK236760	–	–
	MFLUCC 21-0296	*C.moschata*	China	MZ622004	OK236707	OK236612	OK236660	OK236759	–	–
*A.betae-kenyensis*	CBS 118810 ^T^	*Betavulgaris* var. cicla	Kenya	KP124419	KP124270	KP125197	KP124888	KP123966	KP124123	KP124733
*A.breviconidiophora*	MFLUCC 21-0786^T^	*Digitalis* sp.	Italy	MZ621997	OK236698	OK236604	OK236651	OK236751	–	–
*A.burnsii*	CBS 118817	*Tinosporacordifolia*	India	KP124424	KP124274	KP125202	KP124893	KP123971	KP124128	KP124738
	CBS 118816	*Rhizophoramucronata*	India	KP124423	KP124273	KP125201	KP124892	KP123970	KP124127	KP124737
	CBS 130264	Human sputum	India	KP124425	KP124275	KP125203	KP124894	KP123972	KP124129	KP124739
	CBS 879.95	*Sorghum* sp.	UK	KP124422	KP124272	KP125200	KP124891	KP123969	KP124126	KP124736
	CBS 107.38^T^	*Cuminumcyminum*	India	KP124420	JQ646305	KP125198	KP124889	KP123967	KP124124	KP124734
	CBS 108.27	*Gomphrenaglobosa*	Unknown	KC584236	KC584162	KC584727	KC584468	KP123850	KP123997	KP124605
	YZU 191042	*Alliumcepa*	Myanmar	MN656137	MN718663	MN656147	MN656155	MN656142	–	–
	YZU 191003	*A.cepa*	Myanmar	MN656136	MN718662	MN656146	MN656154	MN656141	–	–
	**YZU 231748**	***Helianthusannuus***	**Myanmar**	**OR888998**	**OR963608**	**OR979650**	**PP116480**	**OR979653**	**OR979659**	**PP034180**
	**YZU 231747**	***H.annuus***	**Myanmar**	**OR888996**	**OR963607**	**OR979649**	**PP116479**	**OR979652**	**OR979658**	**PP034179**
*A.falcata*	MFLUCC 21-0123	*Atriplex* sp.	Italy	MZ621992	OK236599	OK236693	OK236649	OK236746	–	–
*A.eichhorniae*	CBS 489.92^T^	*Eichhorniacrassipes*	India	KC146356	KP124276	KP125204	KP124895	KP123973	KP124130	KP124740
*A.ellipsoidialis*	MFLUCC 21-0132	*Eupatoriumcannabinum*	Italy	MZ621989	OK236596	OK236690	OK236643	OK236743	–	–
*A.eupatoriicola*	MFLUCC 21-0122	*E.cannabinum*	Italy	MZ621982	OK236683	OK236589	OK236636	OK236736	–	–
*A.gaisen*	CBS 632.93^R^	*Pyruspyrifolia*	Japan	KC584197	KC584116	KC584658	KC584399	KP123974	AY295033	KP124742
	CBS 118488^R^	*P.pyrifolia*	Japan	KP124427	KP124278	KP125206	KP124897	KP123975	KP124132	KP124743
*A.gossypina*	CBS 102597	*Minneolatangelo*	USA	KP124432	KP124281	KP125211	KP124902	KP123978	KP124137	KP124748
	CBS 104.32^T^	*Gossypium* sp.	Zimbabwe	KP124430	JQ646312	KP125209	KP124900	JQ646395	KP124135	KP124746
*A.iridiaustralis*	CBS 118486^T^	*Iris* sp.	Australia	KP124435	KP124284	KP125214	KP124905	KP123981	KP124140	KP124751
	CBS 118487	*Iris* sp.	Australia	KP124436	KP124285	KP125215	KP124906	KP123982	KP124141	KP124752
	YZU 161003	*Irisensata*	China	MG601454	MG601454	–	MG601456	–	MG601457	–
*A.jacinthicola*	CBS 133751^T^	*Eichhorniacrassipes*	Mali	KP124438	KP124287	KP125217	KP124908	KP123984	KP124143	KP124754
	CBS 878.95	*Arachishypogaea*	Mauritius	KP124437	KP124286	KP125216	KP124907	KP123983	KP124142	KP124753
*A.koreana*	SPL2-1^T^	*Atractylodesovata*	Korea	LC621613	LC621647	LC621715	LC621681	LC631831	LC631844	LC631857
	SPL2-4	*A.ovata*	Korea	LC621615	LC621649	LC621717	LC621683	LC631832	LC631845	LC631858
*A.longipes*	CBS 121333^R^	*Nicotianatabacum*	USA	KP124444	KP124293	KP125223	KP124914	KP123990	KP124150	KP124761
	CBS 540.94	*N.tabacum*	USA	AY278835	AY278811	KC584667	KC584409	AY563304	KP124147	KP124758
*A.minimispora*	MFLUCC 21-0127^T^	*Citrulluslanatus*	Thailand	MZ621980	OK236587	OK236681	OK236634	OK236734	–	–
*A.muriformispora*	MFLUCC 21-0784^T^	*Plantago* sp.	Italy	MZ621976	OK236677	OK236583	OK236630	OK236730	–	–
***A.myanmarensis* sp. nov.**	**YZU 231735**	***Helianthusannuus***	**Myanmar**	**OR888993**	**OR963611**	**OR979651**	**PP508255**	**OR979656**	**OR979662**	**PP034183**
	**YZU 231736^T^**	***H.annuus***	**Myanmar**	**OR897031**	**OR963612**	**OR963615**	**PP508256**	**OR979657**	**OR979663**	**PP034184**
*A.orobanches*	MFLUCC 21-0137^T^	*Orobanche* sp.	Italy	MZ622007	OK236710	–	–	OK236763	–	–
	MFLUCC 21-0303	*Orobanche* sp.	Italy	MZ622008	OK236711	–	–	OK236764	–	–
*A.ovoidea*	MFLUCC 21-0782^T^	*Dactylisglomerata*	Italy	MZ622005	OK236708	OK236614	OK236661	–	–	–
	MFLUCC 21- 0298	*D.glomerata*	Italy	MZ622006	OK236709	OK236615	OK236662	–	–	–
*A.obpyriconidia*	MFLUCC 21-0121^T^	*Viciafaba*	Italy	MZ621978	OK236585	OK236680	OK236633	OK236732	–	–
*A.phragmiticola*	MFLUCC 21-0125^T^	*Phragmites* sp.	Italy	MZ621994	OK236696	OK236602	OK236649	OK236749	–	–
*A.rostroconidia*	MFLUCC 21-0136^T^	*Arabis* sp.	Italy	MZ621969	OK236670	OK236576	OK236623	OK236723	–	–
*A.silicicola*	MFLUCC 22-0072^T^	*Salixalba*	Russia	MZ621999	OK236700	OK236606	OK236653	OK236753	–	–
*A.tomato*	CBS 114.35	*Solanumlycopersicum*	Unknown	KP124446	KP124295	KP125225	KP124916	KP123992	KP124152	KP124763
	CBS 103.30	*S.lycopersicum*	Unknown	KP124445	KP124294	KP125224	KP124915	KP123991	KP124151	KP124762
*A.torilis*	MFLUCC 14-0433^T^	*Torilisarvensis*	Italy	MZ621988	OK236594	OK236688	OK236641	OK236741	–	–
***A.yamethinensis* sp. nov.**	**YZU 231738**	***Helianthusannuus***	**Myanmar**	**OR888995**	**OR963609**	**OR963613**	**PP179252**	**OR979654**	**OR979660**	**PP034181**
	**YZU 231739^T^**	***H.annuus***	**Myanmar**	**OR889008**	**OR963610**	**OR963614**	**PP179253**	**OR979655**	**OR979661**	**PP034182**

Notes: Type strains are marked ‘T’. Representative strains are marked ‘R’. The present strains are in bold.

### ﻿Phylogenetic analysis

The resulting sequences were processed in the GenBank database at the National Center for Biotechnology Information (NCBI) using BLAST searches. The relevant sequences were downloaded and derived from newly reported sequences of recent publications (Woudenberg et al. 2015; Luo et al. 2018; Htun et al. 2022; Li et al. 2022, 2023; Romain et al. 2022) used in the present analysis (Table 2). The adjustments, alignments, and comparative analyses of the gene sequences were executed using ClustalX (Larkin et al. 2007) within the MEGA 11 software platform (Tamura et al. 2021) and gaps were treated as missing data. Maximum-likelihood (ML) and Bayesian inference (BI) methods were utilized to elucidate the phylogenetic relationships among *Alternaria* species. The ML analyses were constructed using the GTRGAMMAI model of nucleotide evolution, and 1000 bootstrap (BS) replicates were performed to assess branch support with RAxML v. 7.0.3 (Stamatakis et al. 2008). Bayesian analysis was conducted with MrBayes v.3.2.6 (Ronquist et al. 2012) with the best-fit model of nucleotide substitution, GTR+I+G, determined by MrModeltest v.2.3 (Posada and Crandall 1998) with the Akaike Information Criterion (AIC). The “MrModelblock” file in MrModeltest was run using both the PAUP path (Swofford 2002) and the MrMt path (Nylander 2004). The two simultaneous Markov Chain Monte Carlo (MCMC) algorithms were launched from random trees, covering 10^6^ generations, with data collected every 100 generations (Rannala and Yang 1996). The analysis was stopped when the standard deviation of split frequencies dropped below 0.01. A burn-in parameter of 25% was established, signifying that 75% of the trees were retained during the burn-in phase, with the remaining trees utilized for calculating the posterior probabilities in the majority-rule consensus tree. Subsequently, the phylogenetic tree was visualized and modified using FigTree v. 1.4.3 (Rambaut 2016). In the phylogram, branch support is indicated by (posterior probability PP/bootstrap value BS) equal to or above 0.6/60%.

## ﻿Results

### ﻿Phylogenetic analyses

**Figure 1.**
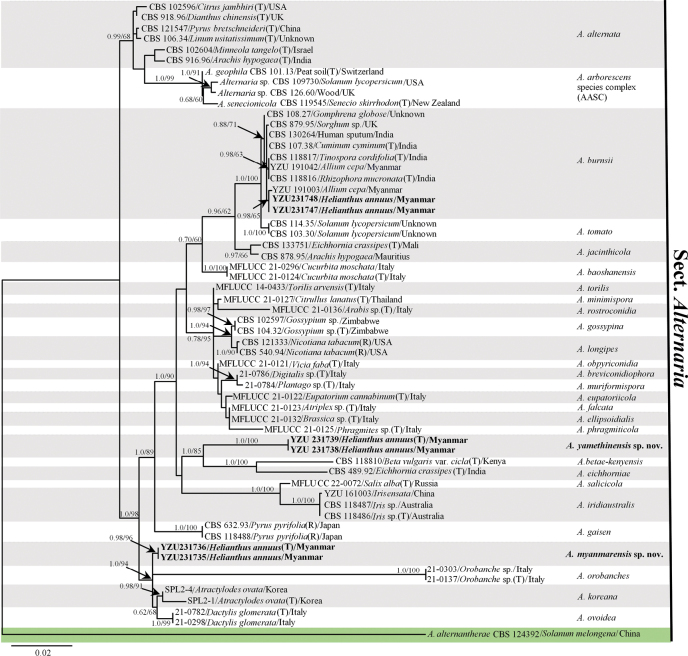
Phylogenetic tree generated from maximum likelihood analyses using aligned ITS, *GAPDH*, *RPB2*, *TEF1*, *Alt a 1*, *EndoPG*, and OPA10-2 gene sequences of the present *Alternaria* strains and their related species. Bootstrap support (BS) values ≥ 60% and Bayesian posterior probability (PP) scores ≥ 0.60 were shown at the nodes (ML/PP). *Alternariaalternantherae* CBS 124392 was used as an outgroup. Type strains are marked ‘T’. Representative strains are marked ‘R’. The strains from the present study are highlighted in bold.

The combined dataset, comprising sequences from seven gene loci (ITS, *GAPDH*, *RPB2*, *TEF1*, *Alt a 1*, *EndoPG*, and OPA10-2), included 59 *Alternaria* strains, containing the present 6 strains. It had 2,722 characters with gaps, allocated as follows: 466 characters for ITS, 302 for *GAPDH*, 307 for *RPB2*, 216 for *TEF1*, 421 for *Alt a1*, 391 for *EndoPG*, and 619 for OPA10-2. The phylogenetic tree was constructed and rooted using *Alternariaalternantherae* CBS 124392 as the outgroup. The Maximum Likelihood (ML) phylogeny was used as the foundational tree. Four strains fell into two independent clades and two, YZU 231747 and YZU 231748, were clustered with the strains of known species *A.burnsii* (Fig. 1). One of the individual clades comprising YZU 231738 and YZU 231739, with PP/BS values of 1.0/100% was found to be sister to *A.betae-kenyensis*, *A.eichhorniae*, *A.iridiaustralis*, and *A.salicicola*. It also fell into a subclade with *A.eichhorniae* and *A.betae-kenyensis* (PP/BS=1.0/85%). Another clade, consisting of YZU 231735 and YZU 231736, exhibited PP/BS values of 0.98/96%, falling into a group with *A.orobanches*, *A.koreana*, and *A.ovoidea*, which is highly supported by PP/BS values of 1.0/94%. Additionally, the strains YZU 231747 and YZU 231748 were clustered with the previously reported *A.burnsii* strains. They also formed a subclade with a strain from Myanmar, YZU 191003, supported by PP/BS values of 0.98/65% (Fig. 1). The results indicated that the current strains represented two new species and a known species of *Alternaria*, all belonging to the section Alternaria.

### ﻿Taxonomy

#### ﻿ Alternaria myanmarensis

Taxon classificationFungiPleosporalesPleosporaceae

M.N. Zin & J.X. Deng sp. nov.

28DC4C6D-0B5F-5C5B-9F3A-797129F8DA58

853961

Fig. 2

##### Etymology.

The specific epithet refers to the location, Myanmar.

##### Holotype.

Myanmar, Mandalay Region, Yamethin Township, Segyi Village (30°21'28.188"N, 112°08'32.136"E), collected from infected leaves of *Helianthusannuus* in August 2023 by Khin Nayyi Htut (YZU–H–2023154, holotype). Ex-type culture (YZU 231736) was also obtained.

##### Description.

**Figure 2.**
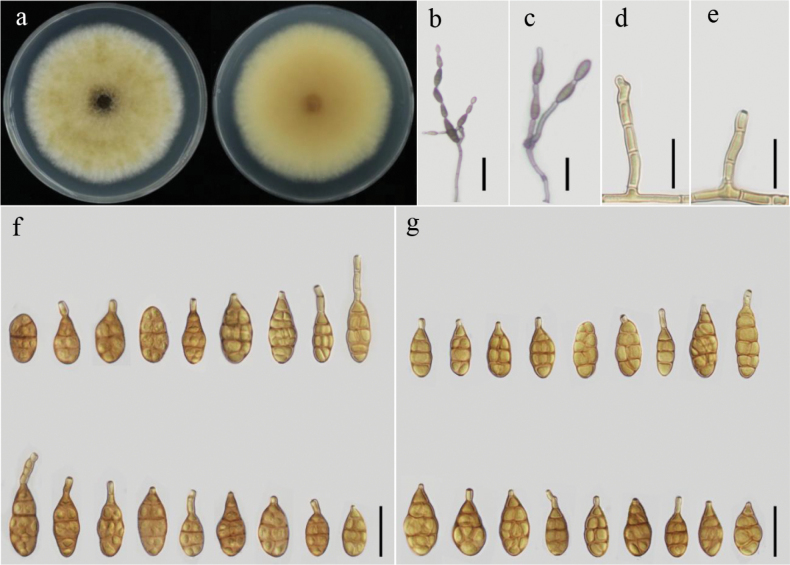
Morphology of *Alternariamyanmarensis* sp. nov. from *Helianthusannuus*: Colony on PDA for 7 days at 25 °C (**a**); Sporulation patterns on V8A (**b**) and on PCA (**c**); Conidiophores on V8A (**d**) and on PCA (**e**); Conidia on V8A (**f**) and on PCA (**g**) at 22 °C. Scale bars: 50 μm (**b, c**); 25 μm (**d–g**).

Colonies on PDA are circular, light vinaceous buff with a white halo at the edge, velvety, cottony, honey to white in reverse, 68–70 mm in diameter (Fig. 2a). On PCA, conidiophores arise directly from lateral or apical aerial hyphae or medium, lightly flexuous, sometimes geniculate at the apex, 27.5–85(–90) × 2–4.5 μm, conidia emerge from the apex or geniculate loci, short to long ellipsoid or narrow-ovoid,10–30(–42) × 7–11 μm, 2–5 transverse septa, 2–6 units per chain, beak 3–12 μm (Fig. 2c, e, g). On V8A, conidiophores arise from near the apex of the terminal hyphae, 24–65(–70) × 3–5 μm, conidia 8–29(–33) × 3–14 μm, 2–5 transverse septa, 3–6 units per chain, beak 1–9 μm (Fig. 2b, d, f).

##### Additional isolate examined.

Myanmar, Mandalay Region, Yamethin Township, Segyi Village (30°21'28.188"N, 112°08'32.136"E) from the infected leaves of *Helianthusannuus*, August 2023, Khin Nayyi Htut, living cultures (YZU 231735).

##### Notes.

This species is phylogenetically grouped with *A.koreana*, *A.orobanches*, and *A.ovoidea*, based on sequences from ITS, *GAPDH*, *RPB2*, *TEF1*, *Alt a 1*, *EndoPG*, and OPA10-2 genes. It is distinct from *A.koreana* and *A.ovoidea* in its smaller conidial body size, particularly in width, and its sporulation patterns which produce catenulate conidia up to 6 units on PCA and V8A media, rather than those of the two closely related species (up to 2 units) on SNA and PDA (Table 3).

**Table 3.** Morphological comparison of the present *Alternaria* and their relevant species.

Species	Conidia				Conidia per chain	Medium	References
	Shape	Body (μm)	Beak (μm)	Septa			
***A.burnsii***	Ovoid or ellipsoid	30–50 × 9–13	–	5–8	Short chain	Host	Simmons (2007)
	Narrow-ovoid or narrow-ellipsoid	30–40 × 8–14	Beakless	3–7	–	PCA , V8A	Simmons (2007)
	Narrow ovoid or ellipsoid	20–50 × 8–15	3–30	4–7	5–9	PCA	Htun et al. (2020)
	**Ovoid or ellipsoid, tapering beak**	**16**–**42** (–**50) × 5**–**15**	**2**–**30** (–**40)**	**2**–**6**	**2**–**6**	**PCA**	**This study**
		**9**–**55** (–**65) × 7**–**12**	**2**–**23** (–**35)**	**2**–**6**	**2**–**9**	**V8A**	**This study**
*A.tomato*	Ellipsoid to long-ovoid	39–65 × 13–22	60–105 × 2	6–9	Solitary	Host	Simmons (2007)
***A.myanmarensis* sp. nov.**	**Short to long ellipsoid or narrow-ovoid**	**10**–**30** (–**42) × 7**–**11**	**3**–**12**	**2**–**5**	**2**–**6**	**PCA**	**This study**
		**8**–**29** (–**33) × 3**–**14**	**1**–**9**	**2**–**5**	**3**–**6**	**V8A**	**This study**
*A.koreana*	Short to long ovoid	12.9–61.2 × 8.6–20.7	–	2–7	1–2	SNA	Romain et al. (2022)
*A.ovoidea*	Ovoid	48–65 × 15.5–30	–	1–3	Solitary	PDA	Li et al. (2022)
*A.orobanches*	Obclavate to ovoid	20–50 × 10–20	–	3–6	1–2	PCA	Li et al. (2023)
***A.yamethinensis* sp. nov.**	**Narrow ovoid or Subellipsoid, blunt-pointed**	**17**–**50** (–**65) × 8**–**14**	**5**–**15 × 2**–**6**	**2**–**7**	**2**–**6**	**PCA**	**This study**
		**32**–**57** (–**63) × 8**–**15**	**1.5**–**8 × 1**–**4**	**2**–**7**	**2**–**9**	**V8A**	**This study**
*A.betae-kenyensis*	Ovoid or subellipsoid	20–28 × 8–10	–	5–7	15–25	PCA	Simmons (2007)
*A.eichhorniae*	Narrow ovoid or subellipsoid, with a blunt-pointed or rounded apical cell	50–70 × 12–18	50–150 × 4–5	7–9	1–2	V8A	Simmons (2007)
*A.iridiaustralis*	Ovoid and short broad ellipsoid	30–40 × 16–24	–	3–4	3–5	PCA	Simmons (2007)
	Ellipsoid or long ellipsoid	20–50 × 15–24	15–100(–133) × 3.5–4.5	1–4	1–2	PCA	Luo et al. (2018)
*A.salicicola*	Straight or curved, subglobose to obclavate or obpyriform	10–50 × 12–38	–	1–6	At least 2	PCA	Li et al. (2023)

#### ﻿ Alternaria yamethinensis

Taxon classificationFungiPleosporalesPleosporaceae

M.N. Zin & J.X. Deng sp. nov.

A8A4C075-E1BF-5118-86ED-302CDDEB35F9

851391

Fig. 3

##### Etymology.

The epithet designation is attributed to the Yamethin township, which was the location where the holotype was originally collected.

##### Holotype.

Myanmar, Mandalay Region, Yamethin Township, Segyi Village (30°21'28.188"N, 112°08'32.136"E) on infected leaves of *Helianthusannuus*, August 2023, Khin Nayyi Htut, (YZU–H–2023154, holotype), ex-type culture (YZU 231739).

##### Description.

**Figure 3.**
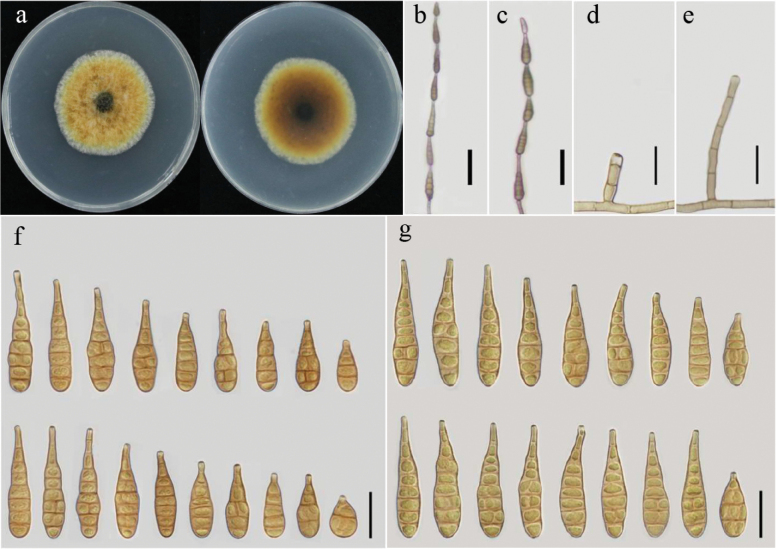
Morphology of *Alternariayamethinensis* sp. nov. from *Helianthusannuus*: Colony on PDA for 7 days at 25 °C (**a**); Sporulation patterns on V8A (**b**) and on PCA (**c**); Conidiophores on V8A (**d**) and on PCA (**e**); Conidia on V8A (**f**) and on PCA (**g**) at 22 °C. Scale bars: 50 μm (**b, c**); 25 μm (**d–g**).

Colonies on PDA are light yellow in the center, white at the edge, with flocculent hyphae, and sulfur yellow to pure yellow in reverse, 38–50 mm in diameter (Fig. 3a). On PCA, conidiophores arise from the substrate, are simple, straight or flexuous, septate, light to brown, 19–85 (–95) × 3–6.5 μm. Conidia arise from the apex or near the apex of the conidiophores, rarely from lateral hyphae, and are narrow ovoid or subellipsoid, blunt-pointed, 17–50 (–65) × 8–14 µm, with 2–7 transverse septa and 2–6 units per chain with a beak 5–15 µm (Fig. 3c, e, g). On V8A, conidiophores are 17–65 (–85.5) × 2–5.5 μm, and conidia are 32–57 (–63) × 8–15 µm with 2–7 transverse septa, 2–9 units per chain and a beak 1.5–8 µm (Fig. 3b, d, f).

##### Additional isolate examined.

Myanmar, Mandalay Region, Yamethin Township, Segyi Village (30°21'28.188"N, 112°08'32.136"E) on infected leaves of *Helianthusannuus*, August 2023, Khin Nayyi Htut, living culture (YZU 231738).

##### Notes.

Phylogenetic analysis based on combined gene regions of ITS, *GAPDH*, *RPB2*, *TEF1*, *Alt a 1*, *EndoPG*, and OPA10-2, along with morphological characteristics, clearly separates this species from others. It can be differentiated from *A.betae-kenyensis* (20–28 × 8–10 µm) by conidial size, *A.eichhorniae* (50–150 × 4–5 µm) and *A.iridiaustralis* (15–100(–133) × 3.5–4.5 µm) by conidial beak, and *A.salicicola* (12–38 µm) by conidial body width. Moreover, it is significantly distinct from those four species by conidial units per chain (Table 3).

#### ﻿ Alternaria burnsii

Taxon classificationFungiPleosporalesPleosporaceae

Uppal, Patel & Kamat, Indian J.Agric.Sci.8:61 (1938)

316F231D-1E03-57B9-B35A-541EEF777D5E

259164

Fig. 4

##### Description.

**Figure 4.**
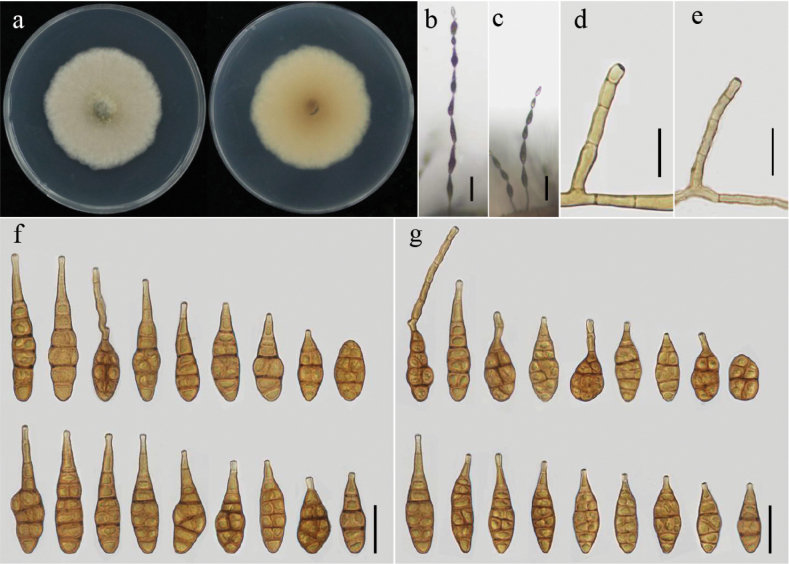
Morphology of *Alternariaburnsii* from *Helianthusannuus*: Colony on PDA for 7 days at 25 °C (**a**); Sporulation patterns on V8A (**b**) and on PCA (**c**); Conidiophores on V8A (**d**) and on PCA (**e**); Conidia on V8A (**f**) and on PCA (**g**) at 22 °C. Scale bars: 50 μm (**b, c**); 25 μm (**d–g**).

Colonies on PDA are dark, surface buff to honey, cottony to vinaceous buff, with a united margin, measuring 62–64 mm in diameter (Fig. 4a). On PCA, conidiophores are single, arising laterally from hyphae, and are either straight or curved, 15–110(–115) × (3–5.5) μm. Conidia emerge from the apex and are ovoid or ellipsoid with a tapering beak, 16–42(–50) × 5–15 μm, with 2–6 transverse septa, 2–6 in a chain, and beaks are 2–30(–40) μm (Fig. 4c, e, g). On V8A, conidiophores 12–95(–103) × (2–4) μm, conidia 9–55(–65) × 7–12 μm, and 2–6 transverse septa, 2–9 in a chain, beaks 2–23(–35) μm (Fig. 4b, d, f).

##### Additional isolate examined.

In Myanmar, Mandalay Region, Yamethin Township, Segyi Village (30°21'28.188"N, 112°08'32.136"E), samples showing disease symptoms on *Helianthusannuus* were collected in August 2023 by Khin Nayyi Htut. The living culture is designated as YZU 231747.

##### Notes.

*A.burnsii* has been found in many countries on different hosts and substrates. The host range of *A.burnsii* is reported to include Apiaceae: *Cuminumcyminum* (Uppal et al. 1938), *Buniumpersicum* (Mondal et al. 2002), *Apiumgraveolens* (Zhang 2003; Zhuang 2005), Cumin (Shekhawat et al. 2013); Cucurbitaceae: *Cucurbitamaxima* (Paul et al. 2015), *Triticumaestivum* and *Phoenixdactylifera* (Al-Nadabi et al. 2018), Coconut (Sunpapao et al. 2022), *Phoenixdactylifera* (Al-Nadabi et al. 2020); Liliaceae: *Alliumcepa* (Htun et al. 2022), and Orchidaceae: *Bletillastriata* (Yin et al. 2023). In the present study, *A.burnsii* was firstly reported from *Helianthusannuus* in Myanmar. Phylogenetically, the present strains fall into a sub-branch with *A.burnsii*YZU 191003 from *Alliumcepa* reported in Myanmar with consistent morphology and nucleotide sequences of ITS, *GAPDH*, *RPB2*, *TEF1*, and *Alt a 1*, gene regions (Htun et al. 2022) (Fig. 1).

## ﻿Discussion

In this study, two new small-spored species, *Alternariamyanmarensis* sp. nov. and *A.yamethinensis* sp. nov., and a known species of *A.burnsii* were identified and illustrated based on morphology and phylogenetic analyses. Molecular research has demonstrated significant separation between large- and small-spored *Alternaria* species (Peever et al. 2004; Hong et al. 2005). The taxonomy of small-spored *Alternaria* species has faced controversies because they exhibit similar morphological characteristics (Wang et al. 2021). Molecular-based assays could facilitate the correct identification alongside morphological traits (Woudenberg et al. 2015; Lawrence et al. 2016). However, molecular analysis has encountered difficulties because the section Alternaria could not be clearly determined using standard markers due to minimal or no variation (Andrew et al. 2009; Prencipe et al. 2023). Previous studies indicated that the identifying criteria of small-spored *Alternaria* become significant only when utilizing a combination of different genes (Woudenberg et al. 2015; Zhang et al. 2023). Agreeing with Romain et al. (2022), the present species are also clearly distinguished based on a multigene sequence analysis, indicating which species belong to the section Alternaria. To date, this section includes more than 91 species, according to recent publications (Gannibal and Lawrence (2016); Nishikawa and Nakashima (2020); Gou et al. 2022, 2023; Li et al. 2023; Liao et al. 2023; He et al. 2024).

Phylogenetically, *A.myanmarensis* sp. nov. and *A.yamethinensis* sp. nov. fall into individual lineages representing new taxa. *A.myanmarensis* sp. nov. is characterized by small conidial body (10–30(–42) × 7–11 μm) catenating in a longer chain (2 to 6 units), compared with its relevant species (solitary or 2 conidia in a chain), *A.koreana* from *Atractylodesovata* in Korea (Romain et al. 2022), *A.orobanches* from *Orobanche* sp. in Italy (Li et al. 2023) and *A.ovoidea* from *Dactylisglomerata* in Italy (Li et al. 2022). Morphologically, *A.yamethinensis* sp. nov. (conidial width 8–14 μm and 2–6 conidial units per chain) is quite different from its closely related species of *A.iridiaustralis* (conidial width 15–24 μm) from *Iris* spp. (Luo et al. 2018), *A.salicicola* (conidial width 12–38 μm) from *Salixalba* in Russia (Li et al. 2023), *A.betae-kenyensis* (15 to 25 conidial units per chain) from *Beta vulgaris* in Kenya (Simmons 2007) and *A.eichhorniae* (solitary or two conidia in a chain) from *Eichhorniacrassipes* in India (Simmons 2007). Additionally, either *RPB2* or OPA10-2 region serves as great marker for the delimitation of the above species.

The genus *Alternaria* ranks 10^th^ among fungal genera for infecting over 4,000 plant species (Thomma 2003). The first record of *Alternariahelianthi* (named *Helminthosporiumhelianthi*) on sunflower in Uganda was done by Hansford (1943). Later, 12 more species were found in various sunflower-growing countries globally, including *A.helianthinficiens* (Simmons 1986), *A.leucanthemi* (Carson 1987), *A.longissima* (Prathuangwong et al. 1991), *A.carthami* (Chowdhury 1994), *A.zinniae* (Bhutta et al. 1997), *A.alternata* (Lagopodi and Thanassoulopoulos 1998), *A.protenta* (Cho and Yu 2000), *A.heliophytonis* (Simmons 2007), *A.roseogrisea* (Roberts 2008), *A.helianthicola* (Rajender et al. 2016), *A.tenuissima* (Wang et al. 2019), and *A.solani* and *A.tomatophila* (Zhang et al. 2021). However, it has been established that *Alternariahelianthi*, which is the synonym of *Alternariasterhelianthi*, was based on morphology and phylogeny (Simmons 2007; Wei et al. 2022). In this study, three *Alternaria* species associated with sunflower in Myanmar have been identified, and pathogenicity tests reveal that these present *Alternaria* species are causal pathogens for sunflower, of which *A.yamethinensis* sp. nov. is identified as the most pathogenic one (Suppl. material 1). This discovery underscores the importance of Alternaria leaf spot and blight on sunflower and helps in disease management in Myanmar.

## Supplementary Material

XML Treatment for Alternaria myanmarensis

XML Treatment for Alternaria yamethinensis

XML Treatment for Alternaria burnsii

## ﻿Additional information

### Author ORCIDs

Zin Mar Nwe https://orcid.org/0009-0000-6376-8306

Khin Nayyi Htut https://orcid.org/0009-0009-9498-8040

Sein Lai Lai Aung https://orcid.org/0009-0006-2738-5598

Ya-Nan Gou https://orcid.org/0009-0005-1740-4065

Cheng-Xin Huang https://orcid.org/0000-0001-7770-5242

Jian-Xin Deng https://orcid.org/0000-0001-7304-5603

### Data availability

All of the data that support the findings of this study are available in the main text or Supplementary Information.

## Supplementary materials

Supplementary material 1 Diseased symptoms of *Helianthusannuus* caused by *Alternaria* spp. Data type doc https://binary.pensoft.net/file/1064643This dataset is made available under the Open Database License (http://opendatacommons.org/licenses/odbl/1.0/). The Open Database License (ODbL) is a license agreement intended to allow users to freely share, modify, and use this Dataset while maintaining this same freedom for others, provided that the original source and author(s) are credited.Zin Mar Nwe, Khin Nayyi Htut, Sein Lai Lai Aung, Ya-Nan Gou, Cheng-Xin Huang, Jian-Xin Deng
