# Supplementary material for: ﻿Two novel species and a new host record of Alternaria (Pleosporales, Pleosporaceae) from sunflower (Compositae) in Myanmar
Source: MycoKeys. 2024 Jun 7;105:337–54. doi: 10.3897/mycokeys.105.123790 (PMC11179095; doi:10.3897/mycokeys.105.123790)
Supplement: Supplementary material 1 — Diseased symptoms of Helianthusannuus caused by Alternaria spp. [file mycokeys-105-337-s001.doc]

**Supplementary material 1**

Diseased symptoms of *Helianthus annuus* caused by *Alternaria* spp.: Diseased symptoms observed in the field (A-D), Pathogenicity test of *Alternaria yamethinensis* sp. nov. (E), *Alternaria myanmarensis* sp. nov. (F), *Alternaria burnsii* (G), and Control (H)
